# Inference of disease associations with unmeasured genetic variants by combining results from genome-wide association studies with linkage disequilibrium patterns in a reference data set

**DOI:** 10.1186/1753-6561-3-s7-s55

**Published:** 2009-12-15

**Authors:** David Hadley, David P Strachan

**Affiliations:** 1 Division of Community Health Sciences, St George's, University of London, London, SW17 0RE, UK

## Abstract

Results from whole-genome association studies of many common diseases are now available. Increasingly, these are being incorporated into meta-analyses to increase the power to detect weak associations with measured single-nucleotide polymorphisms (SNPs). Imputation of genotypes at unmeasured loci has been widely applied using patterns of linkage disequilibrium (LD) observed in the HapMap panels, but there is a need for alternative methods that can utilize the pooled effect estimates from meta-analyses and explore possible associations with SNPs and haplotypes that are not included in HapMap.

By a weighted average technique, we show that association results for common SNPs in an observed data set can be scaled and combined to infer the effect of a genetic variant that has been measured only in an independent reference data set. We show that the ratio *p*(*R*-1)/[1 + *p*(*R*-1)], where *R *is the relative risk associated with a measured or unmeasured allele of frequency *p*, is appropriately scaled by 1/*D*' and weighted in proportion to *r*^2^, both common measures of LD being derived from the reference data set.

We illustrate this computationally simple method by combining the results of a genome-wide association screen from the North American Rheumatoid Arthritis Consortium with LD measures from the British 1958 Birth Cohort, and explore the validity of underlying assumptions about the generalizability of LD from one population to another, and from healthy subjects to subjects with clinical disease.

## Background

The HLA allele DRB1*04 has been shown to be more strongly associated with rheumatoid arthritis than nearby tagging single-nucleotide polymorphisms (SNPs) [1]. We propose here a method of inferring the effects of an unmeasured genetic variant (such as DRB1*04) using linkage disequilibrium (LD) measures from an independent reference data set (the British 1958 Birth Cohort) to scale and weight the associations of rheumatoid arthritis with tagSNPs in the North American Rheumatoid Arthritis Consortium (NARAC) data set, supplied as Problem 1 for the Genetic Analysis Workshop 16.

## Methods

The British 1958 Birth Cohort (B58C) compromises all infants in England, Wales, and Scotland born in one week in 1958. During a follow-up in 2002 to 2004 [2], a cell-line-backed DNA collection was established as a nationally representative reference set for genetic case-control studies. Field protocols and consent forms were approved by the South East England Multi-Centre Research Ethics Committee. Genome-wide data from the Illumina HumanHap550 Beadarray on 1430 members of the B58C was deposited by the Wellcome Trust Sanger Institute [3]. In addition, data on HLA typing using Dynal technologies were deposited by the Diabetes & Inflammation Laboratory, Cambridge [4]. Further details about these deposits and the B58C DNA Collection are published online [5].

The NARAC data set [6], provided as Problem 1 for the Genetic Analysis Workshop 16, consists of individual-level genotype data for 550,000 SNPs tested on the Illumina HumanHap550 Bead Array linked to case/control status for rheumatoid arthritis as well as the HLA alleles at the *DRB1 *locus. We derived a numerical score to represent the number of DRB1*04 alleles for each individual in both the NARAC and B58C data sets. Association tests between the rheumatoid arthritis case/control status and each tagSNP in the MHC region (chromosome 6; 27-33 Mb [7]) were generated using Stata™ 9.2.

We tested whether combining the LD patterns from the B58C and the association patterns from NARAC provide an unbiased estimate the effect of the DRB1*04 allele, using only neighboring tagSNPs (± 300 kb) that are common to both data sets. This application has the advantage that the DRB1*04 allele (our target variant) has been measured directly in both data sets, permitting validity checks between our inferred relative risk estimate and the observed effect in the NARAC case-control study.

We define Φ_obs _= *q*(*R*_obs_-1)/[1 + *q(R*_obs_-1)], where *R*_obs _is the relative risk of disease associated with each copy of a measured allele of frequency *q*. The expression for Φ is similar to that for the population-attributable risk fraction (PARF) in epidemiological studies. However, in this genetic application, because each individual has two chromosomes, the PARF of a variant is Φ(2-Φ). In the Appendix we show that when the tagSNP is more common than the target variant, Φ_obs_/*D*' is an unbiased estimator of Φ_true_, where Φ_true _is the equivalent parameter at the unmeasured target locus and *D*' is the conventional measure of LD between the measured tagSNP and the target variant in an undiseased population. We also show (see Appendix) that, for any given target variant and study design, the variance of Φ_obs _for each tagSNP under the null hypothesis is inversely proportional to the LD measure *r*^2^, relating the measured tagSNP to the unmeasured target variant in the undiseased population.

We calculated a weighted average of the values of Φ_infer _= Φ_obs_/*D*' across all tagSNPs in the selected 600-kb region, and derived an inferred relative risk from this pooled estimate of Φ. In this paper we explore the effect of different assumptions about the generalizability of LD measures and selection of tagSNPs upon this point estimate. An empirical variance of this pooled estimate under the null hypothesis was derived from multiple random permutations of the B58C data set.

## Results

Individually linked *HLA-DRB1 *diplotypes and Illumina HumanHap550 genotypes were available for 1217 members of B58C and for 1187 controls and 799 cases from NARAC. The frequency of DRB1*04 was 21.1% in the B58C reference set, 16.5% in the NARAC controls and 52.2% in the NARAC cases. Counting chromosomes among cases and controls, the observed odds ratio of rheumatoid arthritis per copy of the DRB1*04 variant was 5.54 (95%CI 4.78-6.41) corresponding to a Φ of 0.4280.

All subsequent data relates to 156 SNPs common to both data sets where the minor allele frequency (MAF) of the tag is greater than that of DRB1*04, also less than 44% in the reference data set and |*D*| > 0.01, where *D *is the covariance measure of LD. These restrictions are imposed to remove SNPs where the effect allele could be inconsistent between the B58C data and the NARAC controls data.

**Figure 1:**
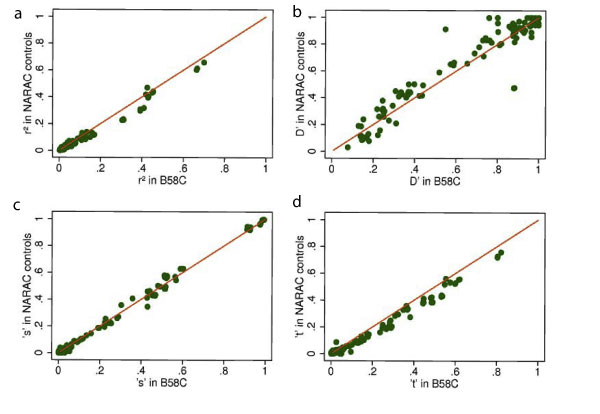
**LD measures in NARAC controls versus B58C**. Clockwise from top left: a) *r*^2^, b) *D*', c) *s*, and d) *t*. See Appendix for description of *s *and *t*.

**Figure 2:**
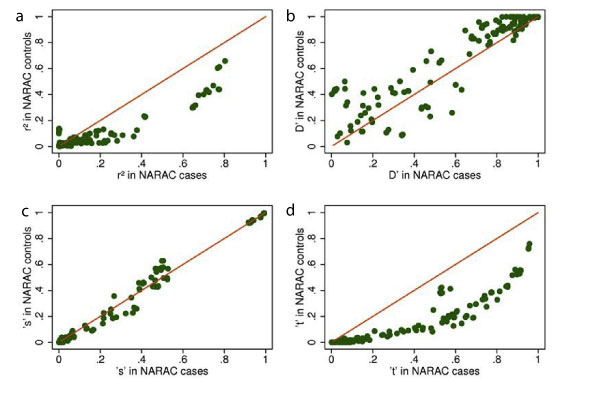
**LD measures in NARAC controls versus case**. Clockwise from top left: a) *r*^2^, b) *D*', c) *s*, and d) *t*. See Appendix for description of *s *and *t*.

For general application, the method needs to apply measures of LD derived from a reference data set to the observed data set where the target variant has not been measured. Figure 1 shows good correspondence for all measures of LD between B58C and NARAC controls. Our inference method assumes that the *s *measure of LD (see Appendix for definition) is constant between cases and controls. In this example, even where there is a strong association between the target variant and disease, this assumption appears valid, as shown in Figure 2. In most situations, the association of the target variant with tagSNPs in the observed case-control data would be unknown and estimated from the reference data set (e.g., B58C). Using *D*' and *r*^2 ^from the B58C data, the pooled estimate of Φ is 0.4530, which is very close to the value of 0.4280 derived directly in the NARAC case-control data.

The variance of the pooled estimate of Φ is greater than the sum of the weights, because many of the neighboring tagSNPs are intercorrelated. The precision of this pooled estimate was estimated by performing 1000 random permutations of the B58C data set to derive the variance of the pooled estimate under the null hypothesis. These results are summarized in Table 1. Inclusion of all 156 SNPs generated a pooled estimate of similar magnitude and precision to that from the three most closely associated SNPs. On the other hand, if no closely associated tagSNPs had been available, results with similar magnitude and only slight loss of precision could be obtained from the majority of SNPs with *r*^2 ^< 0.3. Indeed, in this example, from a region of high LD (*D*' > 0.5), useful information could be derived even from tagSNPs with *r*^2 ^< 0.1.

**Table 1:** Effect of different choices of tagSNP inclusion criteria on estimation of Φ

	Minimum *r*^2^				Maximum *r*^2^			
		
	0	0.2	0.4	0.6	0.1	0.3	0.5	0.7
Pooled Φ	0.453	0.4046	0.4065	0.4253	0.3768	0.5104	0.4569	0.4530
SE of pooled Φ	0.0258	0.0256	0.0256	0.0256	0.0352	0.0292	0.0263	0.0257
Number of tagSNPs used in estimate	156	21	12	3	102	135	153	156

Different combinations of tagSNPs from the NARAC case-control data set, selected by inclusion based on *r*^2 ^between the tagSNPs and the DRB1*04 allele, give slightly different effect estimates (Φ). Also shown is the standard error of Φ under the null hypothesis, derived empirically from 1000 random permutations of the B58C data set.

## Conclusion

Comparing B58C to the NARAC controls, there was close correspondence in LD patterns. Within the NARAC data, however, the LD measures *r*^2 ^and *t *in the region of interest were markedly different in cases and controls, reflecting the strong association between the target variant (DRB1*04) and rheumatoid arthritis. When compared with imputation approaches, the inference method has the advantage of working only with the LD pattern among controls, although it does assume generalizability of the sensitivity parameter *s *from controls to cases. This latter assumption was supported in the NARAC data. The inference approach that we describe can, with modification, be applied to continuous outcomes and offers an alternative to imputation methods that will be particularly attractive for target variants that are not part of standard panels such as HapMap, and for exploring further genome-wide sets of association measures that have been derived from meta-analysis of a particular disease or quantitative trait.

## List of abbreviations used

B58C: British 1958 Birth Cohort; LD: Linkage disequilibrium; NARAC: North American Rheumatoid Arthritis Consortium; PARF: Population-attributable risk fraction; SNPs: Single-nucleotide polymorphisms.

## Appendix

Within a total population, the LD between each tagSNP and an unmeasured target variant may be represented by Table 2, based on relative counts of chromosomes. The parameter *s *represents the proportion of the target variant that also has the tagSNP variant (akin to sensitivity in screening terminology). Another measure, equivalent to the positive predictive value, is *t *(= *sp*/*q*), the proportion of chromosomes with the tagSNP allele that also have the target variant. We seek to infer a value for the true relative risk (*R*) from the measured relative risk (*R*_*obs*_) associated with each tagSNP.

**Table 2:** Haplotype frequencies and disease incidence for variants in positive LD

Measured tagSNP	Target variant (e.g., HLA-DRB1*04)				
	
	Minor allele	Major allele	Relative frequency	Incidence	Derivation
Minor allele	*sp*	*q-sp*	*q*	*I*_*a*_	*I*_*a*_* = I*_*b*_*R*_*obs*_
Major allele	(1-*s*)*p*	1-*q*-(1-*s*)*p*	1-*q*	*I*_*b*_	*I*_*b *_= *I*_*o *_[*R*(1-*s*)*p*+(1-*q*)-(1-*s*)*p*]/[1-*q*]
Relative frequency	*p*	1-*p*	1		
Incidence	*RI*_*o*_	*I*_*o*_		*I*_*total*_	*I*_*tota*_*l *= *I*_*b *_[1+*q*(*R*_*obs*_-1)] *I*_*total *_= *I*_*o *_[1+*p*(*R*-1)]

We define a transformation of *R*, as follows:

We show below that Φ_obs_/*D*' is an unbiased estimator of Φ_true_.

In Table 2: *I*_*b *_= *I*_*o *_[*R *(1-*s*)*p+*(1-*q*)-(1-*s*)*p*]/[1-*q*],

Also in Table 2: *I*_*total *_= *I*_*o *_[1+*p*(*R*-1)] = *I*_*b *_[1+*q *(*R*_*ob *_-1)]

We infer values of Φ_infer _= Φ_obs_/*D*' for each tagSNP and derive a weighted average, with weights inversely proportional to their variance under the null hypothesis. An approximate variance for Φ_infer _can be derived by the Delta method by considering Φ_obs _as a function of *p*, *q*, *D*' and *β *= ln(*R*_*obs*_), as follows, noting that:

Also, if *R *= *R*_*obs *_= 1, and *c *is the proportion of *N *chromosomes that come from cases:

Inverse-variance weights proportional to the LD measure *r*^2 ^are therefore appropriate because *p *(the minor allele frequency for the target variant) is the same for all tag SNPs and *c *and *N *are fixed by the study design.

## Competing interests

The authors declare that they have no competing interests.

## Authors' contributions

DH carried out the data analysis. DPS conceived the method. DH and DPS drafted, read, and approved the manuscript.
